# Large language models generating synthetic clinical datasets: a feasibility and comparative analysis with real-world perioperative data

**DOI:** 10.3389/frai.2025.1533508

**Published:** 2025-02-05

**Authors:** Austin A. Barr, Joshua Quan, Eddie Guo, Emre Sezgin

**Affiliations:** ^1^Cumming School of Medicine, University of Calgary, Calgary, AB, Canada; ^2^The Abigail Wexner Research Institute, Nationwide Children’s Hospital, Columbus, OH, United States; ^3^Department of Pediatrics, The Ohio State University College of Medicine, Columbus, OH, United States

**Keywords:** synthetic data, large language model, artificial intelligence, machine learning, ChatGPT, big data, education, surgery

## Abstract

**Background:**

Clinical data is instrumental to medical research, machine learning (ML) model development, and advancing surgical care, but access is often constrained by privacy regulations and missing data. Synthetic data offers a promising solution to preserve privacy while enabling broader data access. Recent advances in large language models (LLMs) provide an opportunity to generate synthetic data with reduced reliance on domain expertise, computational resources, and pre-training.

**Objective:**

This study aims to assess the feasibility of generating realistic tabular clinical data with OpenAI’s GPT-4o using zero-shot prompting, and evaluate the fidelity of LLM-generated data by comparing its statistical properties to the Vital Signs DataBase (VitalDB), a real-world open-source perioperative dataset.

**Methods:**

In Phase 1, GPT-4o was prompted to generate a dataset with qualitative descriptions of 13 clinical parameters. The resultant data was assessed for general errors, plausibility of outputs, and cross-verification of related parameters. In Phase 2, GPT-4o was prompted to generate a dataset using descriptive statistics of the VitalDB dataset. Fidelity was assessed using two-sample *t*-tests, two-sample proportion tests, and 95% confidence interval (CI) overlap.

**Results:**

In Phase 1, GPT-4o generated a complete and structured dataset comprising 6,166 case files. The dataset was plausible in range and correctly calculated body mass index for all case files based on respective heights and weights. Statistical comparison between the LLM-generated datasets and VitalDB revealed that Phase 2 data achieved significant fidelity. Phase 2 data demonstrated statistical similarity in 12/13 (92.31%) parameters, whereby no statistically significant differences were observed in 6/6 (100.0%) categorical/binary and 6/7 (85.71%) continuous parameters. Overlap of 95% CIs were observed in 6/7 (85.71%) continuous parameters.

**Conclusion:**

Zero-shot prompting with GPT-4o can generate realistic tabular synthetic datasets, which can replicate key statistical properties of real-world perioperative data. This study highlights the potential of LLMs as a novel and accessible modality for synthetic data generation, which may address critical barriers in clinical data access and eliminate the need for technical expertise, extensive computational resources, and pre-training. Further research is warranted to enhance fidelity and investigate the use of LLMs to amplify and augment datasets, preserve multivariate relationships, and train robust ML models.

## 1 Introduction

Clinical data is fundamental to advance medical research and enable the development of machine learning (ML) models. This is particularly relevant in surgical care, as procedural medicine increasingly relies on decision-making based on large-scale data (Maier-Hein et al., 2017). However, access to real-world (real) clinical data is constrained by ethical, legal, and logistical barriers (Pavlenko et al., 2020; Wartenberg and Thompson, 2010). Data requests often require institutional review board approval, data sharing agreements, and compliance with various data privacy regulations (e.g., HIPAA, GDPR, PIPEDA) (Bentzen et al., 2021; Ness, 2007). In many institutions, clinical datasets are proprietary and restricted to internal use. Clinical data also requires significant pre-processing and de-identification procedures which is resource intensive and can delay or hinder research projects—particularly for students, trainees, and early career researchers (Tudur et al., 2017; Willemink et al., 2020). These protections, while essential for patient privacy, limit the accessibility of real clinical data.

In addition to regulatory concerns, clinical data is often incomplete (Newgard and Lewis, 2015). Data collected in clinical settings may suffer from missing values, errors, or biases introduced during data entry. Furthermore, data de-identification processes commonly remove or obscure personal health information (e.g., date of birth, date of operation, demographic data, geographic data). These constraints limit the reliability of analyses as well as the accuracy and generalizability of ML models trained on this data. Collectively, these challenges underscore the need for alternative approaches to provide researchers, learners, and developers with necessary data while preserving patient privacy.

Synthetic clinical datasets, which are artificially generated rather than captured as real patient information, offer a potential solution to the challenges associated with accessing and using real patient data (Beaulieu-Jones et al., 2019; Bellovin et al., 2019; van Breugel and van der Schaar, 2023). Synthetic data can be shared, analyzed, and used freely, bypassing the regulatory and logistical obstacles associated with real data use (El Emam et al., 2020). Despite the potential of synthetic data, achieving high utility, fidelity, and privacy remains a significant challenge (Jordon et al., 2022). Current methods of synthetic data generation, including generative adversarial networks (GANs) (Goodfellow et al., 2020) and variational autoencoders (VAEs) (Kingma and Welling, 2022), have demonstrated utility in synthetic data generation (Goncalves et al., 2020; Jacobs et al., 2023; Rajotte et al., 2022). However, privacy concerns have been raised regarding generative models (Chen et al., 2020; Hayes et al., 2018) including data extractions, model inversions, and membership inference attacks (Rajotte et al., 2022). Furthermore, despite some workarounds, there are issues with mode collapse (Thanh-Tung and Tran, 2020) and the applicability of GANs toward generating categorical and binary data (Jacobs et al., 2023). The use of GANs and VAEs is also self-limiting to those with technical expertise (e.g., complex architecture, fine-tuning) as well as access to necessary computational resources and reference datasets for training.

In recent years, large language models (LLMs)—a computational model capable of language generation and other natural language processing tasks—offer new possibilities for generating text that is coherent and contextually relevant (Brown et al., 2020; Nazir and Wang, 2023). A prominent and publicly available LLM, OpenAI’s ChatGPT, has shown utility in generating synthetic text-based data (Calvo-Lorenzo and Uriarte-Llano, 2024; Hämäläinen et al., 2023; Li et al., 2021). However, the potential of generating tabular synthetic clinical data with ChatGPT remains largely unexplored. Use of LLMs for synthetic data generation may offer an accessible alternative to GANs and VAEs, reducing the need for specialized knowledge and computational resources, which could broaden the reach of synthetic data use in research and ML model development.

This study aims to assess the feasibility of generating realistic tabular clinical data with OpenAI’s GPT-4o (Hurst et al., 2024) using zero-shot prompting, and evaluate the fidelity of LLM-generated data by comparing its statistical properties to the Vital Signs DataBase (VitalDB) (Lee et al., 2022), a real open-source multi-parameter perioperative dataset.

## 2 Methods

### 2.1 Overview

We conducted a two-phase study to evaluate the feasibility of generating synthetic clinical datasets with GPT-4o using a single prompt and without pre-training. In both phases, GPT-4o was prompted to generate a synthetic dataset based on 13 clinical parameters derived from VitalDB. In Phase 1, GPT-4o was prompted with high-level qualitative descriptions of the 13 clinical parameters, to assess its ability to generate a complete and contextually relevant tabular dataset without guiding statistics. In Phase 2, GTP-4o was prompted to generate a synthetic dataset using descriptive statistics of the VitalDB dataset. Both Phase 1 and 2 datasets were statistically compared to VitalDB, with Phase 1 data serving as a baseline for comparisons.

### 2.2 Real dataset

The real clinical dataset used as a comparator in this study is the open-source VitalDB. The VitalDB dataset is a perioperative dataset consisting of multi-parameter data from surgery patients who underwent routine and emergency non-cardiac (general, thoracic, urological, and gynecological) operations at Seoul National University Hospital (Seoul, Korea) from August 2016 to June 2017 (Lee et al., 2022). The dataset included 6,388 de-identified cases encompassing a wide range of clinical parameters including demographic, preoperative, intraoperative, and postoperative parameters.

The VitalDB dataset was selected due to its open-source availability and data completeness for parameters spanning the entire perioperative period. The VitalDB dataset also included a variety of data formats (i.e., numerical, text), variable types (i.e., continuous, categorical, binary), and distributions (i.e., normal, skewed). These considerations ensured a comprehensive evaluation of GPT-4o’s ability to generate and replicate statistical properties of a wide array of clinical data.

### 2.3 Parameter selection and data cleaning

The VitalDB dataset was reviewed for data completeness and parameters with missing data were excluded. Included parameters (*n* = 13) were chosen based on relevance to perioperative care and to represent a range of data formats and variable types. Remaining parameters with similar data formats, variable types, or clinical information were excluded for redundancy in the context of a feasibility study. Timepoint variables in the VitalDB dataset were recorded as the duration from an assigned case start time in seconds. Two time variables were included and converted to hours: operation duration (difference between operation end time and operation start time) and postoperative length of stay (difference between discharge time and operation end time). Selected parameters are presented in Table 1.

**Table 1:** Summary of selected parameters from the VitalDB dataset.

Category (*n*)	Parameters (units)
Demographic data (6)	Case ID, age (years), biological sex (M/F), height (cm), weight (kg), BMI (kg/m^2^)
Preoperative morbidity (3)	ASA physical status classification (1–6), preoperative hypertension (yes/no), preoperative diabetes mellitus (yes/no)
Intraoperative data (3)	Operation type, operation duration (hours), intraoperative transfusion (yes/no)
Postoperative outcomes (1)	Postoperative length of stay (hours)

BMI, body mass index, ASA, American Society of Anesthesiologists.

To further ensure quality and relevance of the data used for comparison, case files for patients younger than 18 (*n* = 57), older than 89 (*n* = 8), missing an American Society of Anesthesiologists (ASA) physical status classification (*n* = 130), and with negative discharge times (*n* = 27) were excluded from the dataset. In total, *n* = 6,166 cases were included.

### 2.4 Generation of synthetic datasets

Prior to the generation of Phase 1 and 2 synthetic datasets, GPT-4o was not pre-trained or provided any patient data from the VitalDB dataset. In Phase 1, GPT-4o was prompted with qualitative descriptions of 13 clinical parameters and asked to generate corresponding data for 6,166 patients. The prompt did not include descriptive statistics, definitions (e.g., ASA physical status classification), or formulas to calculate parameters (e.g., body mass index (BMI)). The prompt used to generate the Phase 1 synthetic dataset is presented in Box 1.

**BOX 1:**
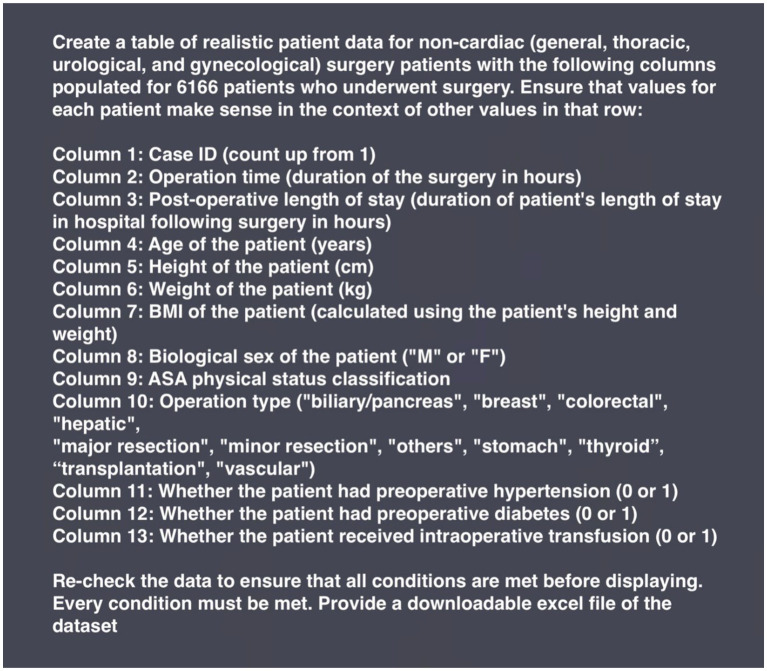
Prompt input to generate the Phase 1 synthetic dataset with GPT-4o.

In Phase 2, GPT-4o was prompted with descriptive statistics of the VitalDB dataset. For continuous parameters (age, height, weight, operation duration, postoperative length of stay), descriptive statistics included mean, standard deviation, and range. Descriptive statistics were not provided for BMI, and GPT-4o was instructed to calculate this parameter using each case file’s corresponding height and weight. Descriptive statistics for height were inputted to GPT-4o as centimeters, requiring the LLM to convert the height parameter to meters in order to calculate BMI—this transformation was not specifically instructed within the prompt. For categorical and binary parameters (ASA physical status classification, operation type, biological sex, preoperative hypertension, preoperative diabetes mellitus, intraoperative transfusion), corresponding proportions were provided. GPT-4o was instructed to assign ascending whole number values for each case ID. For time variables, natural log transformations were used to normalize skewed distributions and GPT-4o was provided with descriptive statistics of the log-transformed values. The prompt used to generate the Phase 2 synthetic dataset is presented in Box 2.

**BOX 2:**
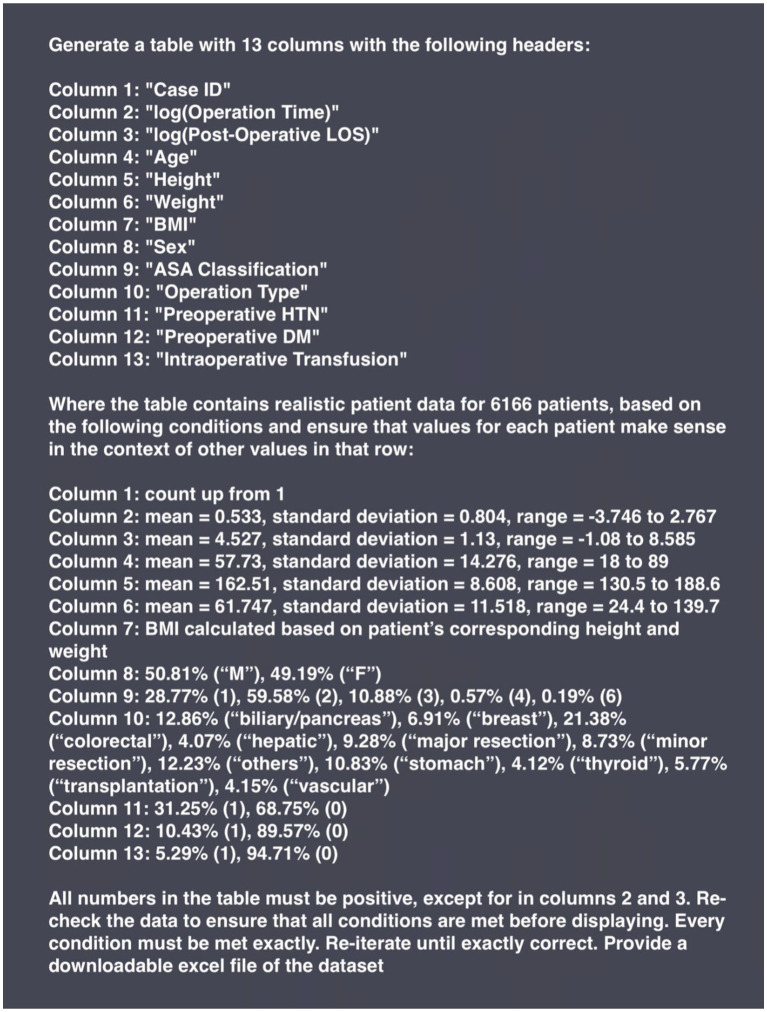
Prompt input to generate the Phase 2 synthetic dataset with GPT-4o.

GPT-4o application programming interfaces (API) were not used in order to determine feasibility of data generation without further technical expertise and resources.

### 2.5 Dataset analysis

The Phase 1 dataset was assessed for general errors, plausibility of outputs, and cross-verification of related parameters. Assessment of general errors evaluated missing data, unexpected outputs, and formatting issues in the tabular data output. Plausibility of outputs involved evaluating time variables for positive values, ASA physical status classification for values between 1 and 6, and categorical and binary parameters for expected values (i.e., only including categories provided in the prompt, category proportions add to 100%). Cross-verification of related parameters involved confirming that all BMI values were appropriately calculated given the corresponding height and weight for each case file.

The Phase 1 and 2 datasets were compared to the VitalDB dataset for statistical similarity. Continuous variables were compared using two-sample t-tests and 95% CI overlap. Given the large sample size, we used parametric two-sample *t*-tests. The log-transformed values of operation duration and postoperative length of stay were used for statistical testing with the two-sample *t*-tests for Phase 2 data. For each continuous parameter, 95% CI overlap was calculated as the proportion of shared values compared to the entire range of values within both 95% CIs from LLM-generated and VitalDB datasets. The Python library Matplotlib was used to generate figures visualizing the overlap of 95% CI for continuous parameters and proportional alignment of categorical and binary parameters. Categorical and binary variables were compared using two-sample proportion tests. Statistical testing was performed using RStudio v.4.4.2 and statistical significance was set at 0.05. For two-sample t-tests and proportion tests, *p*-values above 0.05 indicated statistically insignificant differences in means and proportions, therefore representing an effective replication of descriptive statistical properties from the VitalDB reference dataset.

### 2.6 Ethical considerations

Datasets generated in this study solely represented fictitious patient data. Use of the VitalDB dataset was used in accordance with the requirements outlined by the study team. No data from the VitalDB dataset was inputted directly into GPT-4o for pre-training and no direct data is included in this paper. Furthermore, the synthetic datasets generated by GPT-4o were evaluated solely for research purposes and not used in any form of clinical decision-making.

## 3 Results

In Phase 1, GPT-4o generated a complete and structured tabular dataset comprising 6,166 case files. All 13 expected columns were present, complete, and appropriately labeled; no missing data, unexpected outputs, or formatting issues were present within the generated dataset. Furthermore, all generated time variables were positive, ASA physical status classifications were within the appropriate range (1–6), and categorical and binary parameters only included expected values and proportions all added to 100%. Each case also included a correctly calculated BMI corresponding to the appropriate height and weight.

**Figure 1:**
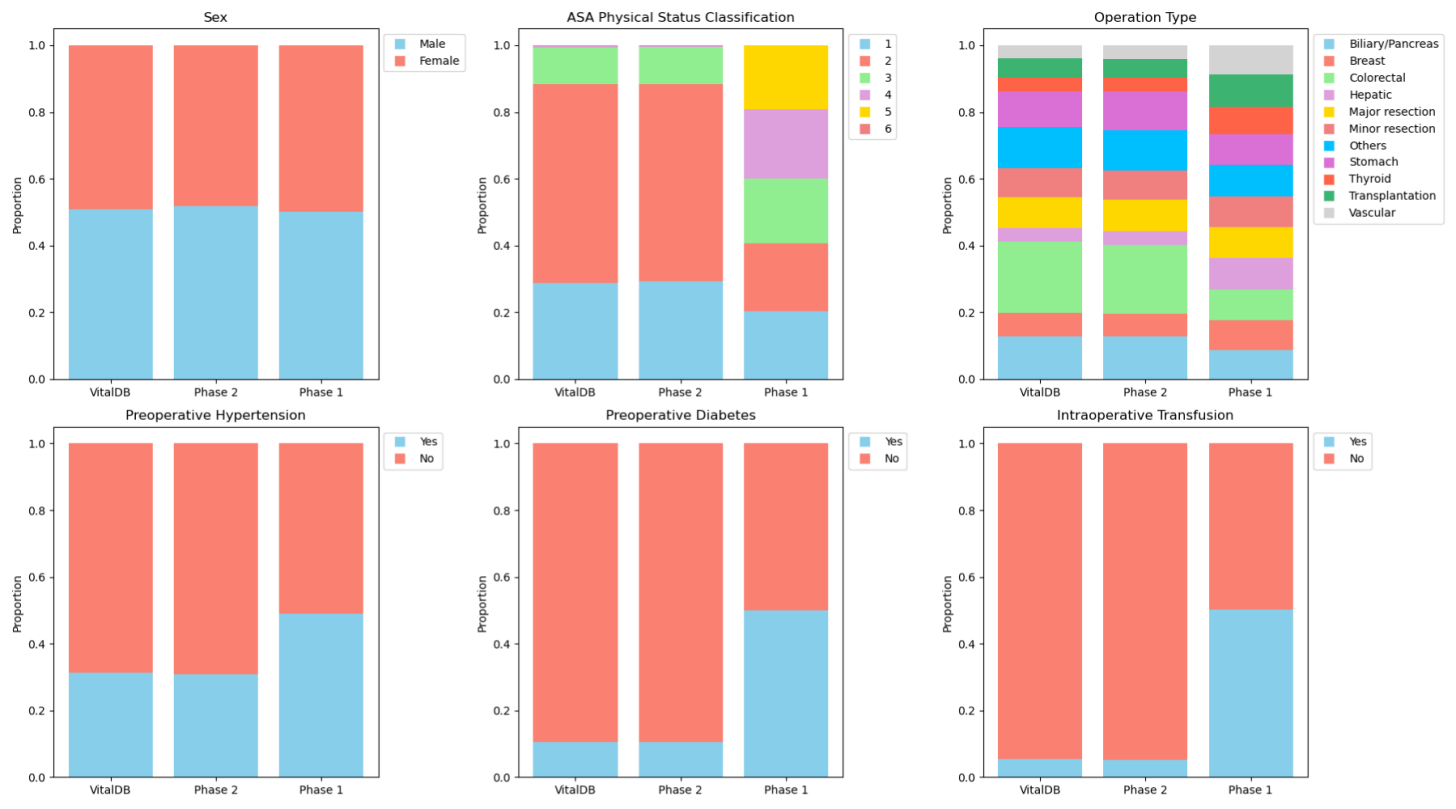
Percent stacked bar plot displaying proportional alignment of categorical and binary parameters between VitalDB, Phase 1, and Phase 2 datasets.

Review of calculated means and ranges for continuous variables included operation duration (6.46 h; 1.00–12.00), postoperative length of stay (154.84 h; 12.00–299.90), age (53.52 years; 18.00–89.00), height (174.51 cm; 150.00–199.00), weight (97.43 kg; 45.00–149.00), and BMI (32.62 kg/m^2^; 11.40–66.20). All continuous parameters showed plausible means and ranges for a perioperative dataset. However, proportions among categorical and binary variables did not differ based on context. Proportions were evenly spread across categories for parameters which are likely to demonstrate uniform distributions (e.g., sex, operation type) as well as parameters that may have skewed distributions (e.g., ASA physical status classification, preoperative comorbidities, intraoperative transfusions). A percent stacked bar plot displaying proportional alignment of categorical and binary parameters can be seen in Figure 1. Overall, generated data in Phase 1 was realistic, displayed appropriate ranges, included correct calculations without the provision of descriptive statistics, formulas, or unit conversions (e.g., BMI), and maintained definitional boundaries of parameters without explicit instructions (e.g., ASA physical status classification).

**Figure 2:**
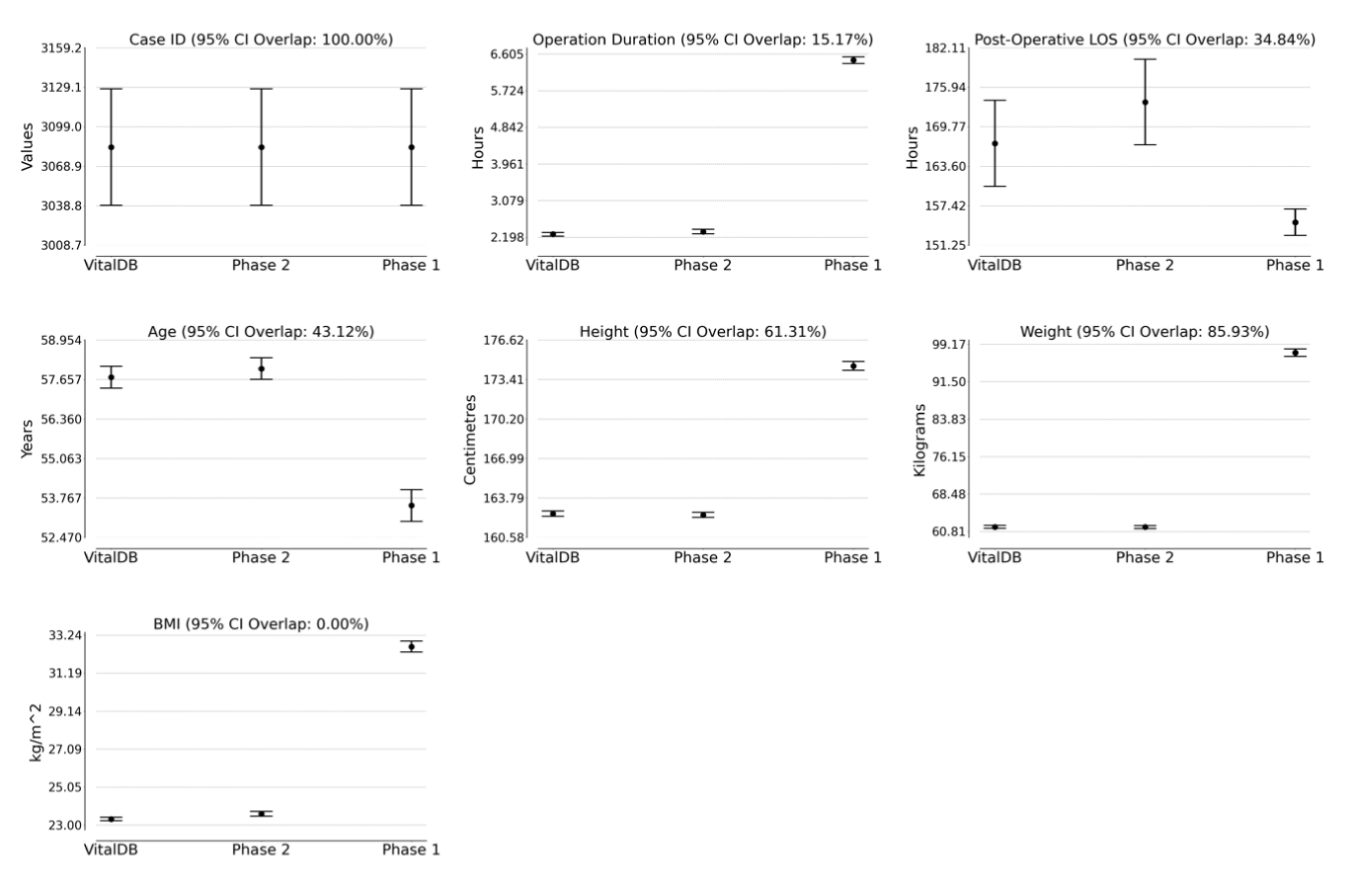
95% confidence intervals for VitalDB, Phase 1, and Phase 2 datasets with labeled percentage of 95% CI overlap between VitalDB and Phase 2 data. LOS, Length of Stay; BMI, body mass index.

The Phase 1 and 2 datasets were statistically compared to the VitalDB dataset. The results of the statistical testing (Tables 2, 3) revealed that 12/13 (92.31%) parameters from the Phase 2 dataset did not show statistically significant differences from VitalDB, including 6/6 (100.00%) of the categorical and binary parameters and 6/7 (85.71%) of the continuous parameters. The only continuous parameter which demonstrated statistically significant differences in Phase 2 was the BMI parameter, which was calculated based on each case’s height and weight rather than generated based on descriptive statistics in the prompt. For the Phase 1 dataset, 2/13 (15.28%) parameters did not show statistically significant differences from VitalDB, one of which was the Case ID parameter. Overlap of 95% CI was observed in 6/7 (85.71%) of the Phase 2 continuous parameters. The measured 95% CI overlaps were as follows: case ID (100.0%), weight (85.93%), height (61.31%), age (43.12%), postoperative length of stay (34.84%), operation duration (15.17%), and BMI (0.0%). The Phase 1 dataset only showed 95% CI overlap in the case ID parameter (100.0%). Visualization of the 95% CI overlaps of each continuous parameter is displayed in Figure 2. Overall, 12/13 (92.31%) of the Phase 2 parameters met the predefined threshold for statistical similarity, demonstrating the parameter effectively replicated statistical properties of corresponding data from the VitalDB dataset.

**Table 2:** Means with associated 95% CI intervals, *p*-values from associated two-sample *t*-tests, and 95% CI overlap values comparing case ID, operation duration, postoperative length of stay, age, height, weight, and BMI between VitalDB and Phase 1 and 2 datasets.

Parameter	VitalDB mean (95% CI)	Phase 1 mean (95% CI)	VitalDB vs Phase 1 *p*-value	VitalDB vs Phase 1 95% CI overlap	Phase 2 mean (95% CI)	VitalDB vs Phase 2 *p*-value	VitalDB vs Phase 2 95% CI overlap
Case ID	3083.5 (3039.06–3127.94)	3083.5 (3039.06–3127.94)	1.000	100.0%	3083.5 (3039.06–3127.94)	1.000	100.0%
Operation duration (hours)	2.27 (2.22–2.31)	6.46 (6.38–6.54)	<0.001	0.0%	2.33 (2.28–2.39)	0.116*	15.17%
Postoperative length of stay (hours)	167.19 (160.48–173.90)	154.84 (152.78–156.90)	<0.001	0.0%	173.6 (166.99–180.31)	0.845*	34.84%
Age (years)	57.73 (57.37–58.09)	53.52 (53.00–54.04)	<0.001	0.0%	58.01 (57.66–58.37)	0.819	43.12%
Height (cm)	162.51 (162.30–162.73)	174.51 (174.16–174.87)	<0.001	0.0%	162.41 (162.20–162.62)	0.200	61.31%
Weight (kg)	61.75 (61.46–62.04)	97.43 (96.67–98.19)	<0.001	0.0%	61.70 (61.42–61.99)	0.327	85.93%
BMI (kg/m^2^)	23.32 (23.23–23.41)	32.62 (32.33–32.91)	<0.001	0.0%	23.60 (23.47–23.73)	0.023	0.0%

*p-values were calculated using log-transformed values.

**Table 3:** Number and proportion of patients by sex, ASA physical status classification, operation type, preoperative hypertension status, preoperative diabetes mellitus status, and intraoperative transfusion status for VitalDB, Phase 1, and Phase 2 datasets with *p*-values from associated two sample proportion tests comparing distributions between VitalDB and synthetic datasets for each parameter.

Parameter	VitalDB *n*, (%)	Phase 1 *n*, (%)	VitalDB vs Phase 1 *p*-value	Phase 2 *n*, (%)	VitalDB vs Phase 2 *p*-value
Sex					
Male	3133 (50.81%)	3087 (50.06%)	0.407	3195 (51.82%)	0.263
Female	3033 (49.19%)	3079 (49.94%)		2971 (48.18%)	
ASA physical status classification					
1	1774 (28.77%)	1249 (20.26%)	<0.001	1810 (29.35%)	0.478
2	3674 (59.58%)	1254 (20.34%)	<0.001	3637 (58.98%)	0.497
3	671 (10.88%)	1198 (19.43%)	<0.001	683 (11.08%)	0.726
4	35 (0.57%)	1291 (20.94%)	<0.001	26 (0.42%)	0.246
5	0 (0.0%)	1174 (19.04%)	<0.001	0 (0.0%)	-
6	12 (0.19%)	0 (0.0%)	<0.001	10 (0.16%)	0.667
Operation type					
Biliary/Pancreas	793 (12.86%)	534 (8.66%)	<0.001	782 (12.68%)	0.764
Breast	426 (6.91%)	558 (9.05%)	<0.001	419 (6.80%)	0.803
Colorectal	1318 (21.38%)	556 (9.02%)	<0.001	1274 (20.66%)	0.332
Hepatic	251 (4.07%)	587 (9.52%)	<0.001	260 (4.22%)	0.682
Major resection	572 (9.28%)	569 (9.23%)	0.928	577 (9.36%)	0.881
Minor resection	538 (8.73%)	563 (9.13%)	0.430	536 (8.69%)	0.952
Others	754 (12.23%)	585 (9.49%)	<0.001	750 (12.16%)	0.912
Stomach	668 (10.83%)	571 (9.26%)	0.004	710 (11.51%)	0.230
Thyroid	254 (4.12%)	509 (8.25%)	<0.001	265 (4.30%)	0.624
Transplantation	356 (5.77%)	596 (9.67%)	<0.001	332 (5.38%)	0.347
Vascular	236 (4.15%)	538 (8.73%)	<0.001	261 (4.23%)	0.254
Preoperative hypertension					
Yes	1927 (31.25%)	3028 (49.11%)	<0.001	1899 (30.80%)	0.582
No	4239 (68.75%)	3138 (50.89%)		4267 (69.20%)	
Preoperative diabetes mellitus					
Yes	643 (10.43%)	3075 (49.87%)	<0.001	645 (10.46%)	0.952
No	5523 (89.57%)	3091 (50.13%)		5521 (89.54%)	
Intraoperative transfusion					
Yes	326 (5.29%)	3098 (50.24%)	<0.001	314 (5.09%)	0.624
No	5840 (94.71%)	3068 (49.76%)		5852 (94.91%)	

The Excel files containing the Phase 1 and 2 synthetic datasets generated with GPT-4o are available in the Supplementary data.

## 4 Discussion

### 4.1 Overview

The present study evaluated the feasibility of generating tabular synthetic clinical data with GPT-4o using zero-shot prompting, and assessed the fidelity of the generated data by comparing it to a real clinical dataset, VitalDB. By examining two phases of data generation—one using qualitative prompts (Phase 1) and another incorporating descriptive statistics from VitalDB (Phase 2)—we explored GPT-4o’s capacity to generate plausible data and replicate statistical properties, variable distributions, and contextual characteristics typical of clinical data. Generated data included various formats (numerical, text), variable types (continuous, categorical, binary), and distributions (normal, skewed), covering demographic, preoperative, intraoperative, and postoperative data. The results indicate: (1) GPT-4o can produce realistic synthetic data without descriptive statistics or reference data, and (2) GPT-4o can generate datasets that align closely with real clinical data, when provided with statistical guidance.

### 4.2 Principal findings and implications

The use of LLMs to generate structured tabular data using zero-shot prompting is a novel concept. Consistent with other modalities of synthetic data generation, LLM-generated data has the potential to address many of the challenges of accessing and using real clinical data (Beaulieu-Jones et al., 2019; El Emam et al., 2020; van Breugel and van der Schaar, 2023). While GPT-4o has yet to demonstrate equivalent fidelity and utility to GANs and VAEs, LLMs may offer solutions to some of their shortcomings. The generation of data using GANs and VAEs requires technical expertise and computational resources. However, data generation using LLMs is accessible to anyone with an internet connection, and can produce clean and ready-to-use datasets, outputted in a downloadable Excel file, through plain-language prompting. This holds substantial implications for democratizing data access in research, educational contexts, and ML model development (Rajotte et al., 2022).

Given that no reference data was required in Phase 1 or inputted for pre-training in Phase 2, LLMs may overcome privacy concerns associated with current approaches to synthetic data generation (Rajotte et al., 2022). The Phase 1 data, which generated realistic clinical data in the absence of guiding statistics, definitions, and formulas, further emphasizes the contextual relevance of outputs from GPT-4o (Brown et al., 2020; Nazir and Wang, 2023). This is particularly useful in educational contexts, whereby learning opportunities for students and trainees can be enhanced by practicing data analysis using synthetic data generated with desired statistical properties and without requiring a reference dataset. LLM-generated datasets can also include synthetic personal health information which would otherwise be removed or de-identified. Since synthetic data can be used without restriction, data can also be re-inputted into LLMs for analysis, which further broadens prospects and scope of future research.

Clean and structured synthetic datasets, with the statistical similarity Phase 2 data demonstrated to VitalDB, has vast implications for data-driven medicine and ML model development. Perioperative data, in particular, is inherently heterogeneous, encompassing a wide variety of sources, formats, and qualities (Maier-Hein et al., 2017). By generating synthetic data which can replicate real-world data distributions, researchers can bypass additional challenges associated with the use of raw clinical data. In this way, synthetic datasets can accelerate the development of predictive tools and surgical decision support systems, ultimately contributing to patient care and surgical outcomes.

### 4.3 Limitations

While this feasibility study demonstrated remarkable preservation of within-column statistical properties and simple relationships between variables, there are some limitations and challenges to consider. First, this study focused solely on GPT-4o, and it remains uncertain whether similar results would be achieved using other LLMs. Similarly, direct comparisons in performance between LLMs, GANs, and VAEs are necessary to assess their relative utility, fidelity, and privacy preservation, which may uncover additional limitations and strengths. At present, it is unclear whether bivariate and multivariate relationships were retained by the LLM-based approach, as this was not directly assessed. Demonstrating the preservation of correlations and other nuanced interdependencies, present in clinical data, is necessary before meaningful comparisons can be made between the performance of LLMs and other data generation methods.

This study also revealed the importance of prompt design in generating accurate and relevant synthetic data outputs. In Phase 1, where prompts lacked descriptive statistics, generated data deviated significantly from the reference dataset. While this underscores the notable results in Phase 2, it also suggests that without explicit guidance, LLMs may produce plausible but statistically unaligned data. Therefore, the quality of outputted data is reliant on effective prompting, and improper prompt design can introduce bias or errors into generated data. It is also unknown whether an iterative approach to prompting may result in greater fidelity.

Continued improvements in LLMs have been previously associated with greater accuracy in a variety of generative and clinically associated tasks (Meyer et al., 2024; Rosoł et al., 2023), and further iterations of GPT-4o may improve upon these results and current limitations.

### 4.4 Future directions

This study’s use of an open-source dataset (VitalDB) as a comparator was intentional to facilitate reproducibility and encourage follow-up research. Future research should continue to investigate the capabilities of LLMs in generating tabular datasets, with particular focus on capturing complex interdependencies between parameters and further assessing reproducibility of results. This should involve using existing and robust frameworks to assess the fidelity and privacy preservation of LLM-generated synthetic datasets (El Emam, 2020; El Emam et al., 2022; Platzer and Reutterer, 2021; Vallevik et al., 2024). Direct comparisons should be made between the performance of GPT-4o and other prominent LLMs. Following further refinement of this zero-shot approach, direct comparisons in utility and privacy should also be conducted between LLMs, GANs, and VAEs, using a systematic benchmarking approach (Yan et al., 2022).

Future work should assess the potential of LLMs in data enhancement, including data amplification and augmentation (El Emam, 2023)—particularly in domains with missing data or limited data availability (e.g., rare diseases, underrepresented patient populations) (Rajotte et al., 2022). By supplementing existing datasets with synthetic data that preserves statistical properties, LLMs could mitigate data scarcity and enable more robust research in data-constrained fields. Applications of LLM-generated synthetic data toward ML model development and validation, predictive tools, and surgical decision support systems should also be explored.

Applications in educational contexts may be evaluated. Surveys, qualitative interviewing, or randomized trials involving students who have used LLM-generated datasets may reveal whether supplementing educational programs with synthetic data can enhance learning for students training toward careers in disciplines which analyze clinical data (e.g., statisticians, data scientists, epidemiologists). It is also recommended to assess whether LLMs can be used to effectively summarize and analyze outputted synthetic data.

## 5 Conclusion

This study demonstrates that zero-shot prompting with GPT-4o can generate realistic tabular synthetic datasets that replicate key statistical properties of real perioperative data. By eliminating the need for technical expertise, extensive computational resources, and pre-training in synthetic data generation, LLMs can offer an accessible modality to address critical barriers associated with clinical data access. Collectively, these findings highlight the broad implications of LLM-generated synthetic data in democratizing data access and enhancing educational opportunities. Future research should focus on enhancing fidelity and investigating the application of LLMs in data amplification and augmentation, replication of multivariate relationships, and ML model development.

## Data Availability

The original contributions presented in the study are included in the article/Supplementary material, further inquiries can be directed to the corresponding author.
